# King Edward's Hospital Fund for London

**Published:** 1910-03-12

**Authors:** 


### KING EDWARD'S HOSPITAL FUND FOR LONDON.

### ANNUAL REPORT.

The annual meeting of the President and General Council of King Edward's Hospital Fund for London, to receive the accounts and the report of the General Council for the year 1909, was held at Marlborough House on Thursday, March 3, His Royal Highness the Prince of Wales, President of the Fund, being in the chair.

Lord Rothschild (the Honorary Treasurer) in presenting the account of receipts and expenditure and the balance-sheet for the year ended December 31, 1909, said he would, with His Royal Highness' permission, venture to make just two remarks. The amount of receipts in 1909, including additions to capital, was £258,926 3s. 2d., a considerable amount, although less than the amount received in some years, and the expenditure was £153,000. Since the end of the year they had so far received £31,000, as against £30,000 last year, and he had also learnt from the newspapers that sums amounting to about £30,000 had been left to the Fund. Owing to the constitution of the Fund now, all moneys given or left without any specific direction could only be invested in trust securities, but if the donor or testator wished them to be invested in non-trust securities that could be done. In that way they held at the present moment a considerable amount of non-trust securities, and the effect of that was that the King's Hospital Fund benefited to the extent of £16,000 a year more from their investments in non-trust securities than would have been the case if the moneys had been invested in trust securities.

The Prince of Wales formally moved the adoption of the accounts, which was seconded by Lord Strathcona, who said the result of the working of the Fund was an even better one than last year, and he was sure that His Royal Highness and all concerned might heartily congratulate themselves upon it.

The resolution having been supported by Sir Henry Burdett, was then put and carried unanimously.

### The Council's Report.

Sir Savile Crossley (Honorary Secretary) read the draft report of the Council for the year 1909, as follows :—

#### Receipts.

The total receipts for the year 1909 were £258,926 3s. 2d. This sum was made up as follows: Donations, £12,145 6s. 11d.; contributions to capital, £279 3s. 10d., together with £13,782 5s. 8d. net profit on realisation of securities; annual subscriptions, £23,615 2s. 7d.; contribution of the League of Mercy, £19,000; from the Lewis Estate (exclusive of interest), £75,000; legacies (including interest on Lewis legacy), £46,401 18s. 5d.; interest from investments, £68,502 5s. 9d.; together with £200 from the trustees of the Bawden Fund.

#### Grants.

The amount distributed was £150,000, being an increase of £10,000 over the sum distributed in 1908. For the first time therefore the sum mentioned by his present Majesty at the inauguration of the Fund has been attained. The Council desire to express their gratitude to all those whose contributions, large or small, have made it possible to achieve this result, and they trust they may receive the public support necessary to maintain this rate of distribution. The total sum distributed amongst hospitals, convalescent homes, and consumption sanatoria since the foundation of the Fund now exceeds £1,000,000, the exact figure (after deducting grants to the extent of £4,659 16s. 7d. that have been allowed to lapse) being £1,134,916 8s. 5d. The Council desire to express their thanks to the trustees of the London Parochial Charities, who for eleven years terminating in 1908 placed at their disposal the sum of £1,000 for distribution amongst convalescent homes, and thus enabled the Fund to undertake this branch of its work.

#### Administration Expenses.

Since the inauguration of the Fund the amount spent on administration has been £1 4s. 7d. per cent. of the total amount received.

#### Legacies, Donations, and Subscriptions.

During the year the final instalment of £5,000 was received from the executors of the will of the late Mr. Samuel Lewis on account of the legacy of £250,000 left to the Fund, together with interest amounting to £8,058 16s., and a first payment of £70,000 on account of the share of the residue of the estate. Amongst other legacies received were the following: £9,000 from the late Sir George Livesey; £6,837 16s. 6d., being the balance of the residue of the estate of the late Lieut.-Gen. G. E. Baynes; £5,000 from the late Miss E. S. Wolfe; £3,000 from the late Rev. W. H. Cooper; and £2,000 from the late Mr. Ellis Franklin. The Fund has also to acknowledge an award of £7,348 17s. 2d. made by the High Court, being the residue of the estate of the late Mr. J. T. Bell, and a donation of £4,775, being one-half of the present available surplus of the Franco-British Exhibition, handed over to the Secretary of State for Foreign Affairs and placed by him at the disposal of the Fund. The amounts received in donations and subscriptions, excluding gifts to capital, show, as compared with 1908, an increase of £3,090.

#### League of Mercy.

The League of Mercy has again contributed £19,000. During the eleven years of its existence the League has furnished in all £135,000 to the Fund. The Council desire to record their grateful appreciation of the labour of the voluntary workers who have contributed towards the collection of this large sum.

The King's and Prince of Wales' Interest.

His Majesty the King, our patron, has been pleased to maintain the deep interest in the Fund which he has taken from its inception. His Royal Highness the Prince of Wales has continued to exercise personal supervision over the work of the Fund. In an appendix to this report will be found an account of the proceedings of the General Council meetings on December 13, 1909, and March 3, 1910, at which His Royal Highness presided.

Expenses of Charity Entertainments.

In May 1909 the Executive Committee, with the approval of His Royal Highness the President, appointed a sub-committee, consisting of Lord Richard Cavendish (chairman), Mr. Griffiths, and Mr. Sydney, with Mr. Fry, to consider the question of the expenses of charity entertainments. The sub-committee reported that while there was no evidence of widespread extravagance or abuse in this connection, certain precautions were desirable. This report was circulated amongst the hospitals both with a view to assisting them to frame regulations for their own protection and as an indication of the readiness of the Fund to deal with the subject if desired.

The Fund's Visitors' Work.

Every eligible hospital and consumption sanatorium which applied for a grant was inspected and reported upon by the Fund's visitors, consisting as heretofore of one medical and one layman in each case.

Statistics of Expenditure.

The annual statistical report prepared by the Fund, which had previously been printed for private circulation only, has this year been published and placed on sale. The report now deals with the expenditure on in-patients and out-patients respectively at 66 hospitals, and the Council hope that the wider distribution of the report may increase public interest in the efforts which are being made in the direction of economy.

Hospital Reconstruction or Extension.

The Distribution Committee have continued to make a careful examination of all schemes for the extension or reconstruction of hospitals, submitted in accordance with the request of the Council. The Fund thus uses its best endeavours to ensure that when capital expenditure of this kind is undertaken, due attention is paid to economy, efficiency, the prospect of funds for future maintenance, and the relation of each scheme to the general question of hospital accommodation in London.

The Throat, Nose, and Ear Hospitals.

The Council observe that the negotiations for the amalgamation of the throat, nose, and ear hospitals have made no progress during the year. While the Fund has again set aside for this purpose a sum considerably in excess of the probable maintenance grants to the separate institutions, the amounts set aside in 1906 and 1907 have now lapsed in accordance with the conditions attaching to grants which remain unapplied for two years.

Consumption Sanatoria.

The Council have approved of a proposal by the Convalescent Homes Committee to enter into negotiations with consumption sanatoria in the country with a view to securing beds which could be placed at the disposal of hospitals in London for the accommodation of suitable cases urgently requiring country treatment. It is hoped that during 1910 more than one scheme of this kind will be definitely arranged.

Committee of Inquiry into Out-patients System.

In the last report it was stated that His Royal Highness the President had announced his intention of appointing a committee to inquire into the system prevailing in the London voluntary hospitals with regard to the admission of out-patients, but that the actual appointment would be deferred until after the Royal Commision on the Poor Laws had issued its report. The whole of the evidence taken by that Commission on the subject of medical relief was not available at the end of the year 1909, but it is hoped that the proposed Committee of Inquiry will be appointed in the course of the year 1910.

Auditors.

The President of the Local Government Board, in pursuance of his powers under the Act, has appointed Messrs. Deloitte, Plender, Griffiths and Co. to audit the accounts of the Fund for the year.

Thanks to the Press.

The Council are obliged to the Press for the services they have rendered in the past, and continue to rely upon their kindly co-operation in obtaining for the Fund wider publicity and support.

The Prince's Speech.

The Prince of Wales, in moving the adoption of the report, said : My Lords and Gentlemen,—It is always a great pleasure to me to preside at our annual meeting, and all the more so when I move the adoption of so satisfactory a report as that presented to us to-day. As the distribution meeting of the Council in December is the recognised occasion for reference to the events of the year, I will only now mention two points which seem to me of special interest in the report. I think we are justified in congratulating ourselves upon the fact that the total sum distributed among hospitals, convalescent homes, and sanatoria since the foundation of the Fund, thirteen years ago, now exceeds one million sterling, the exact figures being £1,134,916 8s. 5d. I would like to refer to the satisfactory list of legacies received during the past year, amounting to £46,400, not including £75,000 from the estate of the late Mr. Samuel Lewis, from which latter source the Fund has benefited to the extent of £328,000. As I said in December, with reference to the Committee of Inquiry into the system prevailing in the London voluntary hospitals with regard to the admission of out-patients, I hope that it may be appointed during this year, and commence work at the beginning of 1911. I have now much pleasure in moving the adoption of the report. (Hear, hear.)

The Duke of Norfolk, in seconding the motion, said he was sure they all echoed the expression of His Royal Highness's satisfaction that the Fund had now reached the point that His Majesty had in his mind and in his heart when he first promoted it. That was a matter of the keenest satisfaction to all of them.

The Rev. T. Bowman Stephenson said he very heartily supported the resolution. It seemed to him that the Fund had conquered, so to speak, its proper position among charities and that it was becoming the very good habit of many in considering the disposal of their fortunes to set aside some fair portion for this great and wonderful Fund. It also seemed to him that the Fund was exercising a most healthy influence upon hospital administration. Whilst they did not attempt to interfere with the internal management of hospitals, yet in a quiet way and a way least likely to give offence, hospital administration was being continually improved.

The resolution was then put and carried unanimously.

The Vote of Thanks.

The Speaker of the House of Commons (the Right Hon. James W. Lowther, M.P.), in moving a vote of thanks to the Prince of Wales for presiding, said that they might all congratulate themselves upon the marvellous success which had been achieved by the Fund, as shown by the report which had been so well received and which had created so much satisfaction amongst them all. He was sure that he was not only expressing the opinion of all in that room, but he believed also of all the inhabitants of the Metropolis, when he said how much they were indebted to His Royal Highness for the great personal interest which he had shown in the Fund, and that if it had not been for that element so great a success would not have been achieved by this time, if ever. He concluded by expressing, on his own behalf, and he was sure on behalf of all, their sincere thanks to His Royal Highness, both for the great interest he had always taken in the Fund, and for presiding over the meeting of the Council that day. (Hear, hear.)

The vote having been seconded by the Master of Elibank and carried by acclamation, His Royal Highness said : My Lords and Gentlemen,—I wish to express my warmest thanks for the kind vote of thanks which you have just passed.

